# Semiquantitative microscopic pore characterizations of the metamorphic rock reservoir in the central paleo-uplift belt, Songliao Basin

**DOI:** 10.1038/s41598-022-05960-y

**Published:** 2022-02-16

**Authors:** Zhouqiang Zeng, Xuanlong Shan, Guoli Hao, Wentong He, Changqing Zheng, Jian Yi, Jiannan Guo

**Affiliations:** 1 Key Laboratory for Evolution of Past Life and Environment in Northeast Asia (Jilin University), Ministry of Education, Changchun, 130012 China; 2 College of Earth Science, Jilin University, Changchun, Jilin, 130061 People’s Republic of China

**Keywords:** Energy science and technology, Fossil fuels, Natural gas

## Abstract

Currently, metamorphic rock is a common target for natural gas exploration, and reservoirs are the key factors restricting natural gas exploration and development in metamorphic rocks. The deep metamorphic rock gas reservoir in the central paleo-uplift of the northern Songliao Basin has good exploration and development potential. In this study, we use a combination of qualitative descriptions and quantitative analysis to comprehensively analyze the pore characteristics of the reservoir and explore the factors controlling the pore characteristics of the metamorphic rock reservoir in the central paleo-uplift belt of the Songliao Basin. The metamorphic rock reservoir in the central paleo-uplift belt contains three types of lithologies: chlorite schist, mica schist and mylonite, each with different protoliths and metamorphic histories. The results of high-pressure mercury intrusion and nitrogen adsorption indicate that the pore size distributions of the schist and mylonite differ. Compared with the mylonite, the schist has larger reservoir space, more heterogeneity, smaller pore size, larger specific surface area and larger adsorbed gas storage capacity. This paper also studies the formation process of the reservoir and divides it into four stages. Finally, this article discusses in detail the factors controlling the microscopic pore characteristics of metamorphic rock reservoirs in the central paleo-uplift belt; the metamorphic rock protolith is the most important controlling factor.

## Introduction

Currently, metamorphic rock is a common target for natural gas exploration, and reservoir properties are the key factors restricting natural gas exploration and development in metamorphic rocks^1,2^. It is now generally believed that the most important reservoir space in metamorphic rock reservoirs is fractures^3–6^. Therefore, through core observation and imaging logging, many scholars have finely characterized fractures, including the length, width, density, opening, dip of fractures, and the angle between ground stress and the strike of fractures (to judge whether the fracture is easy to open during fracturing), and have studied the formation, evolution stages and classification of fractures^1–3,5,7–9^. At present, research on fractures in metamorphic rock reservoirs is relatively mature^3,9^. It is undeniable that fractures have a very important impact on the gas production of metamorphic rock reservoirs. However, in addition to fractures, there are many pores and microfractures that are invisible to the naked eye and cannot be identified by image logging in metamorphic rock reservoirs^8,10^. Similar to shale, these microscopic pores and microfractures can also become reservoir spaces and flow channels for natural gas, and fracturing technology makes production of natural gas from smaller pores possible^11^. Therefore, it is necessary to study the microscopic pores in metamorphic rock reservoirs.

The pore characteristics of the metamorphic rock reservoir are relatively underexplored^12^. At present, research on metamorphic rock reservoirs is mainly based on fracture characterization and well logging identification, and there are few studies on the pore characteristics of metamorphic rock reservoirs and the factors that control them^6,9^.

Considerable controversy continues over the factors controlling the pore structure of metamorphic rock reservoirs. Xia et al. propose that the degree of metamorphism has a great impact on reservoir properties^10^, and Zhu et al. suggest that the lithology of metamorphic rocks is a core factor controlling reservoir formation and the preservation of reservoir space^13^. Han et al. suggest that the main factors controlling metamorphic reservoir development are temperature, lithology, minerals, dissolution, faults, and paleogeomorphology^1^. Liu et al. believe that the formation of dissolution pores in metamorphic rock reservoirs is closely related to the presence of meteoric water, organic acids, and deep fluids^14^.

Previous studies pay little attention to the pore characteristics of metamorphic rock reservoir, and most of them use qualitative methods. In this way, the understanding of the reservoir space of metamorphic rock reservoir is not comprehensive, and only the combination of microscopic pores and macroscopic fractures can provide a more comprehensive understanding of the reservoir space in metamorphic rock reservoirs. Therefore, in this study, we selected the cores of metamorphic rock reservoirs with underdeveloped macroscopic fractures. Based on the traditional qualitative description (thin section analysis, thin sections and scanning electron microscopy), quantitative analysis (high-pressure mercury intrusion and nitrogen adsorption) is added. In this way, The pore characteristics are clearly characterized, which provides a basis for the selection of exploration direction and development measures of metamorphic rock gas reservoirs.

## Geological setting

The Songliao Basin is an important petroliferous basin in northeastern China that has three major structural sequences: a depression sequence, a faulted sequence, and the basement. A large number of oil fields, represented by the Daqing Changyuan superlarge oilfield, have been discovered in the depression sequence; and a large number of natural gas fields, represented by the Xujiaweizi gas field, have been discovered in the faulted sequence^15,16^. However, the exploration level of hydrocarbon resources in the basement of the Songliao Basin is still very low and is still in its infancy.

**Figure 1:**
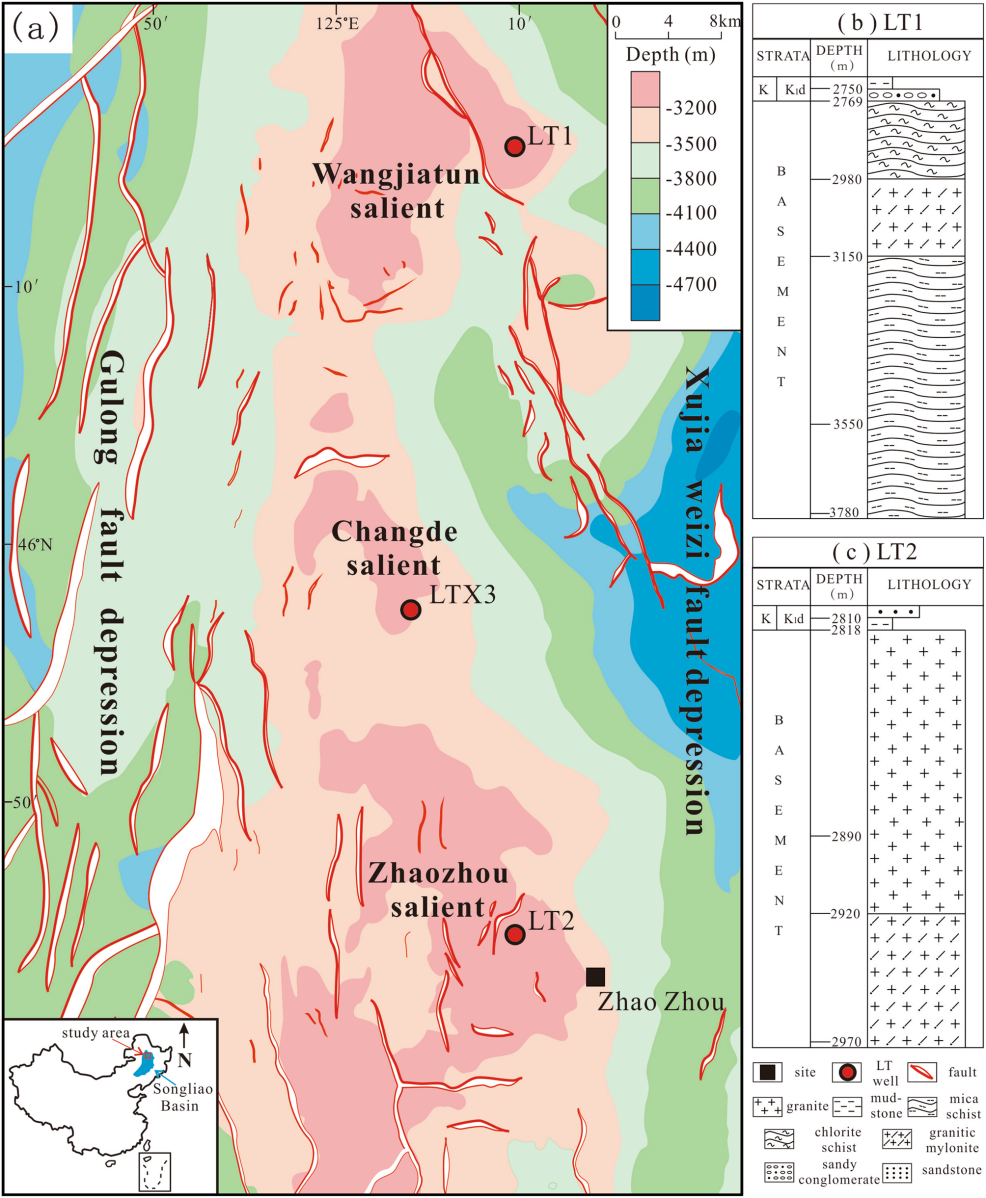
(**a**) Structural map of the top of the basement in the central paleo-uplift zone and the distribution of well locations (modified from Du et al., 20,171); (**b**) basement stratigraphic column of the LT1 well; (**c**) basement stratigraphic column of the LT2 well (This figure was drawn by CorelDRAW Graphics Suite 2019, vision number: 21.3.0.755, url:https://www.corel.com/cn).

In the three main salients in the central paleo-uplift belt, namely, the Wangjiatun salient, Zhaozhou salient and Changde salient, three risk wells were developed: the LT1, LT2 and LTX3 wells (Fig. 1a). Among them, wells LT1 and LT2 reveal a metamorphic rock reservoir in the basement and have good natural gas shows. The daily gas production rate of the metamorphic rock reservoir in the LT1 well is 1.0 × 10^4^ m^3^, and that of the LT2 well is 3.6 × 10^4^ m^3^ ^16^, which shows that the basement of the central paleo-uplift belt has good exploration potential and is an important strategic area for hydrocarbon resources in the Songliao Basin^17^.

The gas accumulation pattern of metamorphic reservoir in central paleo-uplift belt is shown as a “late gas generation in old reservoir” type, and its gas source is mainly Cretaceous Shahezi Formation source rock in Xujiaweizi fault depression. Two types of source rocks, dark mudstone and coal seam, are developed in Shahezi Formation. The average TOC content of dark mudstone is 1.57%, the average content of chloroform asphalt "A" is 0.049%, and the average content of R_o_ is 2.89%; The average TOC content of coal seam is 43.66%, and the average content of chloroform asphalt "A" is 0.2307%. The kerogen type is mainly type III. The organic matter abundance of source rock is high, which has reached high maturity-over maturity, and is a very good gas source rock^17^.

The investigated districts are located in the central paleo-uplift belt, which is located between the Xujiaweizi fault depression and the Gulong fault depression in the north (Fig. 1a) and is a buried hill-like uplift zone formed on a basement containing thrust folds. The strata below the second member of the Denglouku Formation are absent above the basement, and the Denglouku Formation directly overlaps it.

## Petrological characteristics

In this study, a total of 8 metamorphic rock reservoir samples were collected from LT1 (Fig. 1-b) and LT2 (Fig. 1-c) wells for further analysis. According to the thin section identification and the mineral composition content (Table 1) determined by X-ray diffraction (XRD), the lithology of three types of metamorphic rocks is revealed.

**Table 1:** Mineral composition of metamorphic rocks.

Sample ID	Well	Depth (m)	Quartz	Potash feldspar (%)	Plagio-clase (%)	Mus-covite (%)	Biot-ite (%)	Epid-ote (%)	Garn-et (%)	Total Clay (%)
1	LT1	2778.67	17.9	1.3	50.2		7.7	4.4		18.5
2	LT1	2780.5	31.9		40.7	4.6		0.7		19.6
3	LT1	3374.44	28.7	0.6	17.7	10.8		8.0		33.8
4	LT1	3444.33	37.1		25.6	4.1			1.3	24.2
5	LT1	2986.97	45.9	6.7	29.8	5.9				8.5
6	LT1	2989.77	44.9	4.8	29.9	6.0				10.2
7	LT2	2961.7	36.9	24.3	27.3		1.0	4.5	1.7	1.6
8	LT2	2964.4	35.5	21.7	33.2		1.3	1.6	1.9	1.8

### Chlorite schist

**Figure 2:**
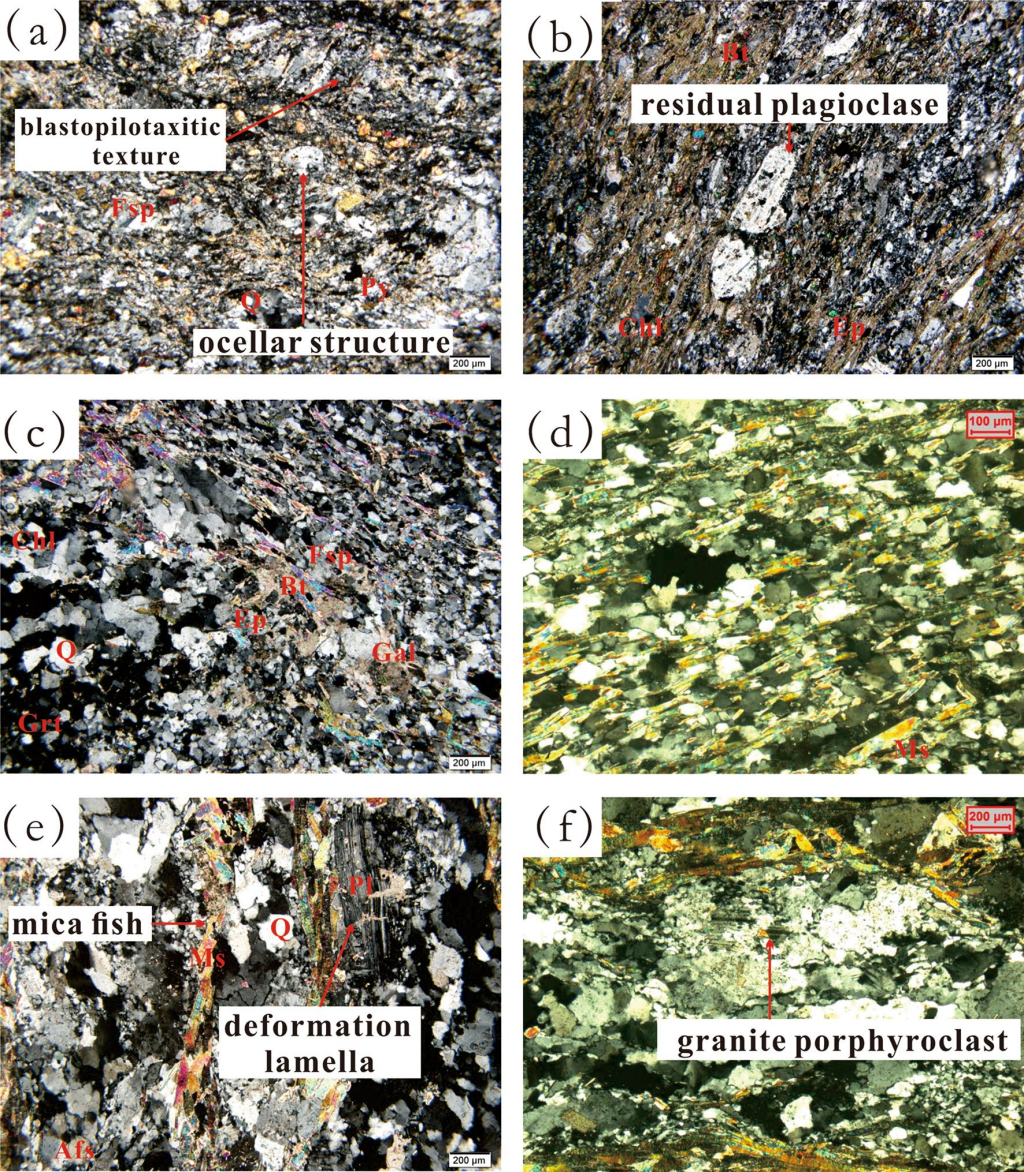
Photomicrographs showing the petrological characteristics of the metamorphic rock reservoir: (**a**) sample 1, chlorite schist (−); (b) sample 1, sample 2, chlorite schist ( +); (c) sample 4, mica schist ( +); (**d**) sample 3, mica schist (−); (**e**) sample 5, mylonite ( +); (**f**) sample 7, mylonite ( +).

The chlorite schist (Samples 1 and 2) has a blasto-pilotaxitic texture (Fig. 2-a) and schistose structure (Fig. 2-b) and is mainly composed of quartz, feldspar, chlorite, biotite, epidote and calcite. The minerals show directional alignment. Residual andesite phenocrysts and residual plagioclase can be observed (Fig. 2-b); albites have an ocellar structure (Fig. 2-a). Therefore, the protolith of chlorite schist is andesite that experienced dynamic metamorphism (Table 2).

**Table 2:** Lithology, protolith and metamorphism of samples.

Sample ID	Well	Lithology	Protolith	Metamorphism
1	LT1	Chlorite schist	Andesite	Dynamic
2	LT1	Chlorite schist	Andesite	Dynamic
3	LT1	Mica schist	Clastic rock	Regional
4	LT1	Mica schist	Clastic rock	Regional
5	LT1	Mylonite	Granite	Dynamic
6	LT1	Mylonite	Granite	Dynamic
7	LT2	Mylonite	Granite	Dynamic
8	LT2	Mylonite	Granite	Dynamic

### Mica schist

The mica schist (Samples 3 and 4) has a porphyritic crystalloblastic texture, fine granular lepidoblastic texture and schistose structure and is mainly composed of quartz, feldspar, chlorite, mica (including biotite and muscovite) and garnet (Fig. 2-c). Quartz and feldspar grains are separated by directionally aligned biotite and muscovite. Faint blastopsammitic structure (Fig. 2-d) can be observed. The abundance of micas implies that the rock contained mud, so the protolith was a clastic rock, and the metamorphism was regional (Table 2).

### Mylonite

The mylonite (Samples 5, 6, 7, and 8) has a blastogranitic texture, deformation lamella and mica fish (Fig. 2-e) and is mainly composed of quartz, plagioclase, potash feldspar and muscovite. Perthite porphyroclasts (Fig. 2-f) can be observed, and their edges consist of fine quartz that has been dynamically recrystallized and oriented, retaining the granitic structure of the protolith. The feldspar grains are subhedral, the quartz grains are allotriomorphic, and there are no signs of transport. In summary, the protolith was a granite, and the metamorphism was dynamic (Table 2).

## Methods

### Field emission scanning electron microscopy (FE-SEM)

Field emission scanning electron microscopy (FE-SEM) experiments were carried out at the Test Science Experiment Centre of Jilin University. The microstructural morphology of the metamorphic rocks was observed using JSN-6700F equipment. The samples were cut into slices with a length and width of 1 cm and 1 mm thickness and subjected to ion polishing. All images were obtained under a high vacuum mode with an 8 kV voltage. The working distance was approximately 10 mm.

### High-pressure mercury intrusion (HPMI)

High-pressure mercury intrusion (HPMI) experiments were conducted with a Micromeritics AutoPore IV 9520 system at the Beijing Centre for Physical and Chemical Analysis. The maximum intrusion pressure was 30,000 psia (206.8 MPa), corresponding to a pore throat diameter of 6.6 nm. The experimental process was as follows: samples were loaded into the core chamber and vacuum pumped for 1 h. After mercury had filled the core chamber, measuring valves and balance valves were opened. Then, mercury was injected gradually under an applied pressure to measure the capillary pressure. Once a maximum pressure of 30,000 psia was reached, the pressure started to decrease progressively, and mercury was extruded from the sample. Capillary pressure curves were derived by Micromeritics AutoPore IV 9520 software. The pore size distribution (PSD) was calculated according to the Washburn equation. The laboratory procedure followed Standard GB-T 21650.1-2008.

### Nitrogen adsorption experiments

Nitrogen adsorption experiments were carried out at the Test Science Experiment Centre of Jilin University. Prior to adsorption measurements, all metamorphic rock samples were prepared by sieving to a size of 50–80 mesh and then weighing 2–6 g per sample. The samples were dried at 200 °C for 6 h in a vacuum oven to remove moisture and volatile gases from the rocks. The Micromeritics ASAP2020 automatic device was used in the experiment, measuring the gas adsorption volume over the relative equilibrium adsorption pressure (P/P_0_) range of 0.01–0.99, and the determinations of adsorption isotherms and outgassing were carried out in a liquid nitrogen environment.

## Results

### Microscopic pore types and morphological characteristics

**Figure 3:**
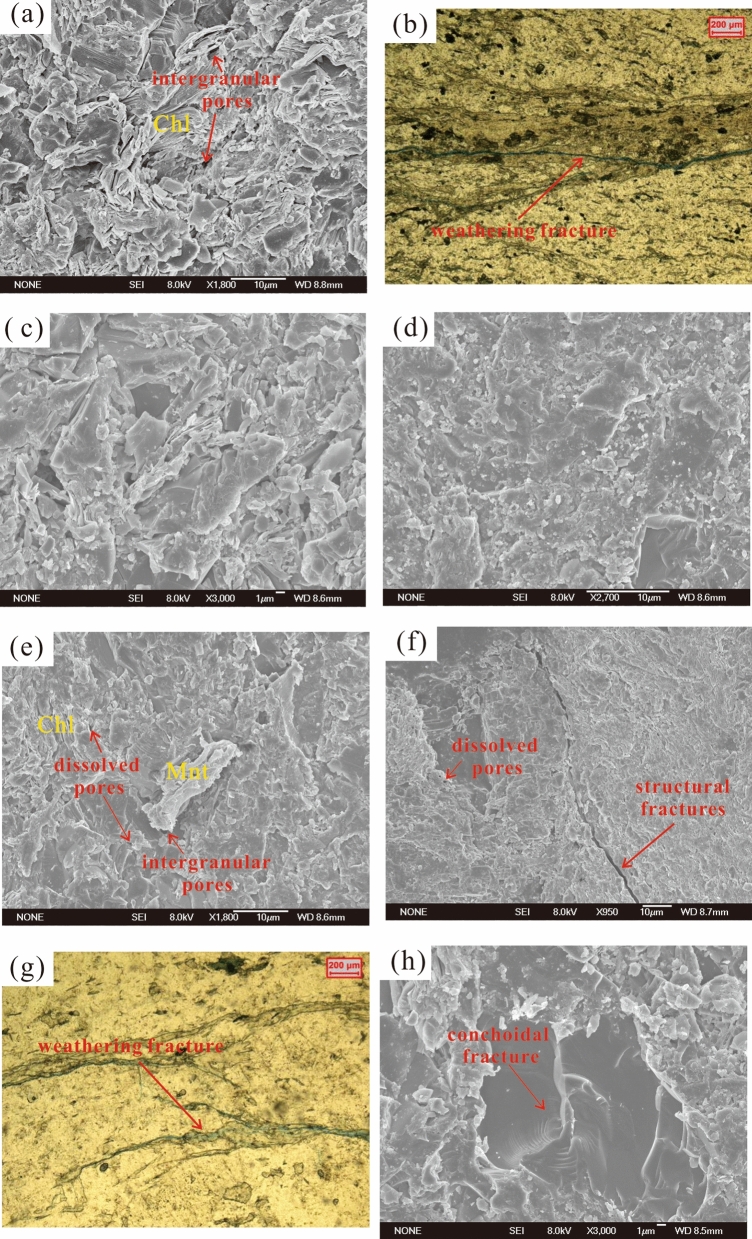
Types of reservoir space in metamorphic rock samples. (**a**) chlorite schist, 1; (**b**) chlorite schist, 2; (**c**) chlorite schist, 2, with quartz surface; (**d**) mica schist, 3, with quartz surface; (**e**) mica schist, 3; (**f**) mylonite, 6; (**g**) mylonite, 5; (**h**) mylonite, 7, with quartz surface.

Clay minerals can be identified by scanning electron microscopy^18,19^. The chlorite schist contains many chlorites (Fig. 3-a), and the mica schist has the most types of clay minerals, including biotite, chlorite and montmorillonite (Fig. 3-e), while mylonite has only a small amount of chlorite.

Figure 3-c, 3-d and 3-h are all quartz particle surfaces. V-shaped holes can be observed in the chlorite schist, and the quartz particles are largely broken (Fig. 3-c), indicating that the chlorite schist formed mainly by mechanical effects during the process of rock formation. The mica schist has little evidence of mechanical effects during the process of rock formation, and although V-shaped holes can be seen (Fig. 3-f), the number of holes is small. Many scales are warped and separated from the carrier and have a tendency to spall (Fig. 3-d). Although these scales may also be debris formed in the grinding process of thin sections, their number is much greater than those in the chlorite schist (Fig. 3-c) and mylonite (Fig. 3-h), and the scales are small; thus, they can be considered scaly spalling (a typical characteristic of chemical dissolution effects^20^). Irregular dissolution pits are also observed, so the mica schist was mainly chemically dissolved during the process of rock formation. The mylonite has obvious conchoidal fractures (Fig. 3-h); therefore, mechanical effects were clearly predominant during the process of rock formation.

Based on previous studies^10^ on the classification of metamorphic rock reservoir space and this study, the reservoir space of the metamorphic rock reservoir in the central paleo-uplift belt can be divided into the following four types: intergranular pores, dissolved pores, structural microfractures and weathering microfractures (Table 3). The main reservoir space in the chlorite schist is in the form of intergranular pores (Fig. 3-a) and weathering microfractures (Fig. 3-b); the main reservoir space in the mica schist is in the form of intergranular pores and dissolved pores (Fig. 3-e); and the main reservoir space in the mylonite is in the form of dissolved pores, structural microfractures (Fig. 3-f) and weathering microfractures (Fig. 3-g).

**Table 3:** Types and characteristics of reservoir spaces in the metamorphic rock reservoir from the central paleo-uplift belt of the Songliao Basin.

Reservoir space types	Origin	Characteristics
Intergranular pores	Primary intergranular pores in protolith and pores formed among metamorphic mineral	Angular, with a small pore size, visible under a scanning electron microscope
Dissolved pores	Leaching, dissolution by formation fluid	Generally development along fractures, irregular shape
Structural microfractures	Microfractures formed under tectonic stress	Irregular, extending far
Weathering microfractures	Formed by physical weathering and breaking along the schistosity plane	Linear, funnel-shaped, developed in schist

### Characteristics of high-pressure mercury porosity

**Figure 4:**
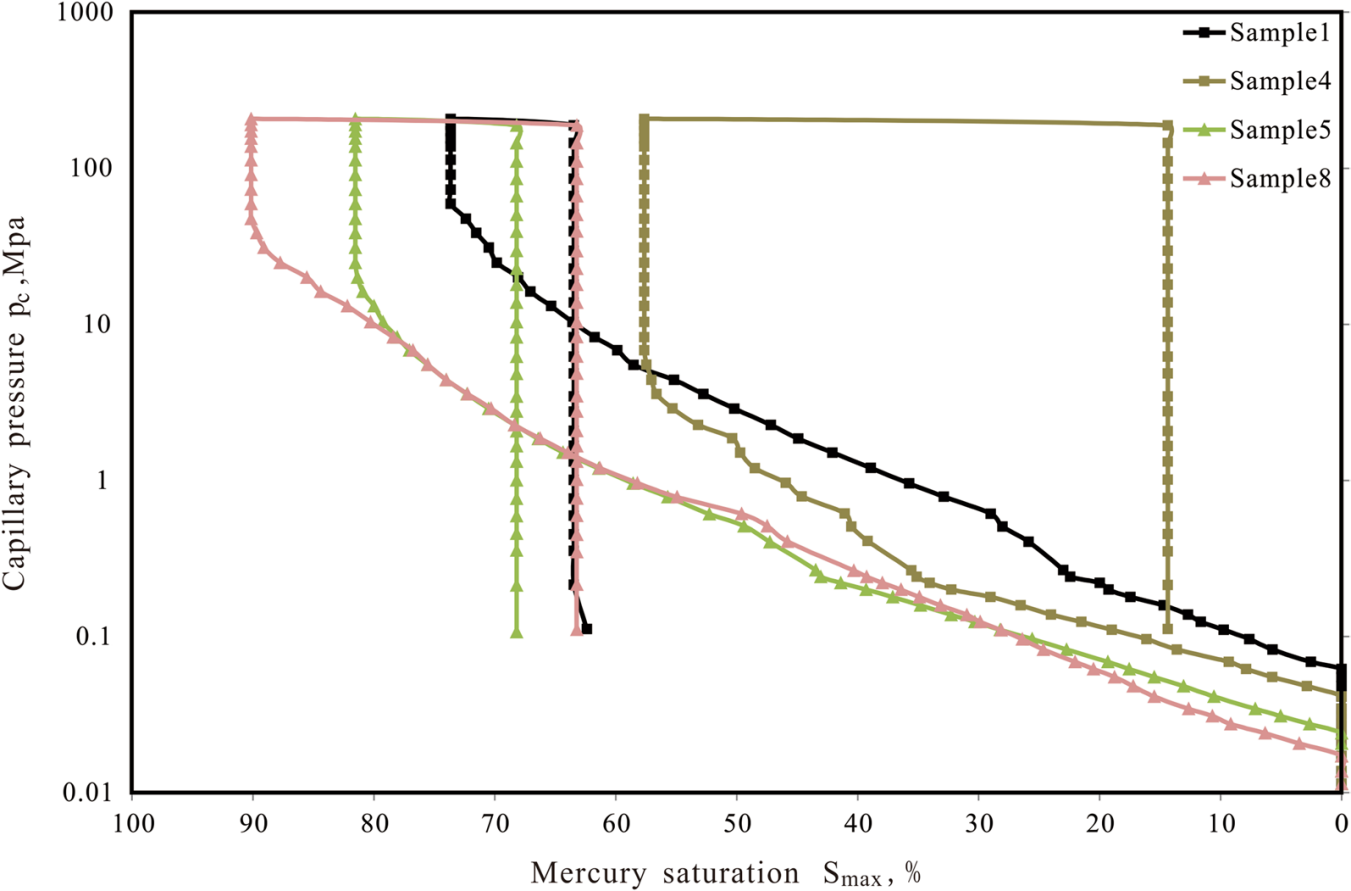
Capillary pressure curve of the metamorphic rock reservoir sample.

HPMI have proven to be effective methods for characterizing the pore structures of porous media^21^. Figure 4 presents the high-pressure mercury capillary curves of schist and mylonite. The curve is generally linear, indicating poor pore sorting. The calculated mercury intrusion parameters are shown in Table 4. The average porosity of schist is 1.46%, the average porosity of mylonite is 1.00%, and the porosity of schist is higher than that of mylonite.

**Table 4:** Parameters of high-pressure mercury intrusion for metamorphic rock samples.

Sample ID	Porosity (%)	Displacement pressure (MPa)	Maximum pore throat (μm)	Withdrawal efficiency (%)
1	1.51	0.0643	19.40	11.25
2	1.98	0.0275	45.32	7.68
3	1.63	0.0370	33.72	6.43
4	0.73	0.0412	29.77	43.28
Schist Average	1.46	0.0425	32.05	17.16
5	1.28	0.0255	48.87	13.34
6	1.07	0.0241	51.75	20.54
7	0.91	0.0240	51.93	23.48
8	0.73	0.0192	64.85	26.91
Mylonite Average	1.00	0.0232	54.35	21.07

The displacement pressures of the metamorphic rock samples are very low. For the schist, the maximum value is 0.0643 MPa, and the average value is 0.0425 MPa. The maximum displacement pressure of the mylonite is only 0.0255 MPa, and the average is 0.0232 MPa (Table 4), which is significantly less than that of the schist, indicating that the mylonite has a larger maximum pore throat and higher permeability^22^. The maximum mercury saturation can reflect the heterogeneity of the reservoir. The maximum mercury saturation of schist is 73.64% on average, while that of the mylonite is 84.32% on average (Table 4), reflecting that the schist is more significant heterogeneous and that mylonite is more significant homogeneous. The retreat mercury curves of the schist and mylonite are linear, and a large amount of mercury is withdrawn at the first point of pressure reduction. After this point, there is very little mercury withdrawal. The withdrawal efficiencies are very low, with values of 30.25% for the schist and 24.84% for the mylonite on average (Table 4).

**Figure 5:**
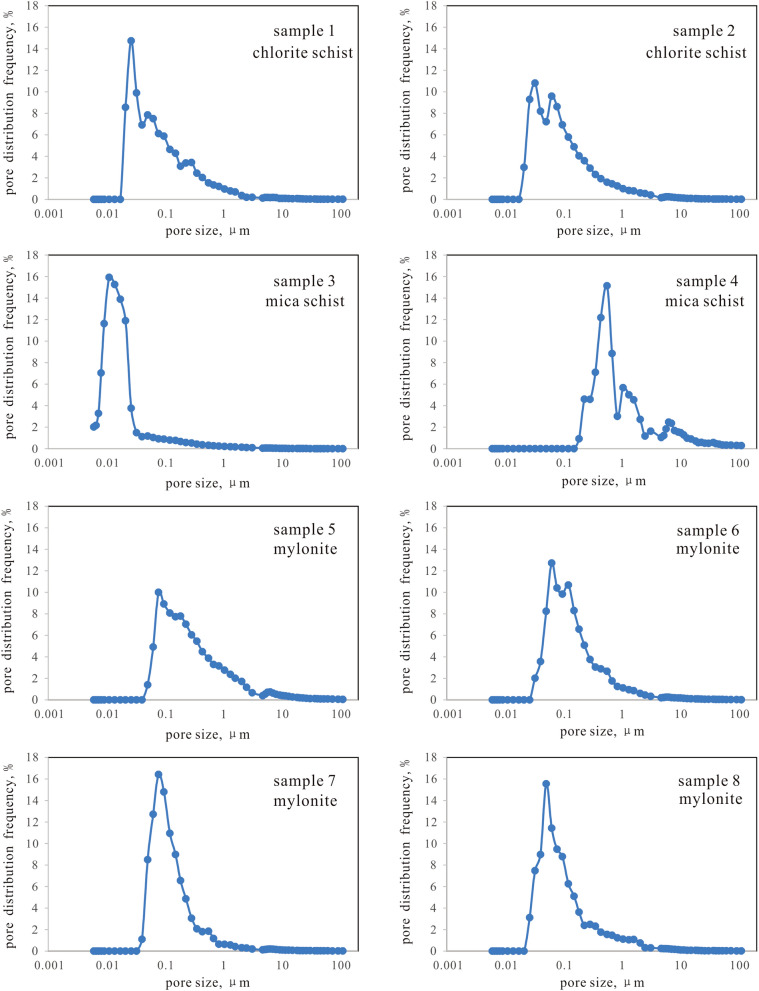
Pore distributions of metamorphic rock reservoir samples through high-pressure mercury injection.

The pore distribution of metamorphic rock reservoir samples is shown in Fig. 5. The pore size distribution of 8 samples showed a unimodal distribution. The porosity of chlorite schist less than 0.1 micron accounts for more than 60%. The pore size of mylonite is mainly distributed in the range of 0.1–1 micron and less than 0.1 micron (Table 5).

**Table 5:** Volume distribution of HPMI.

Sample ID	Distribution frequency (%)			
	> 10 μm	1-10 μm	0.1-1 μm	< 0.1 μm
1	0.77	4.36	27.41	67.47
2	0.97	5.56	29.78	63.69
3	0.23	1.26	4.98	93.52
4	10.66	32.95	56.39	0
5	3.01	14.78	56.98	25.24
6	1.33	5.87	46.02	46.79
7	0.90	3.57	41.99	53.54
8	0.91	6.06	28.22	64.81

### Characteristics by nitrogen adsorption experiments

**Figure 6:**
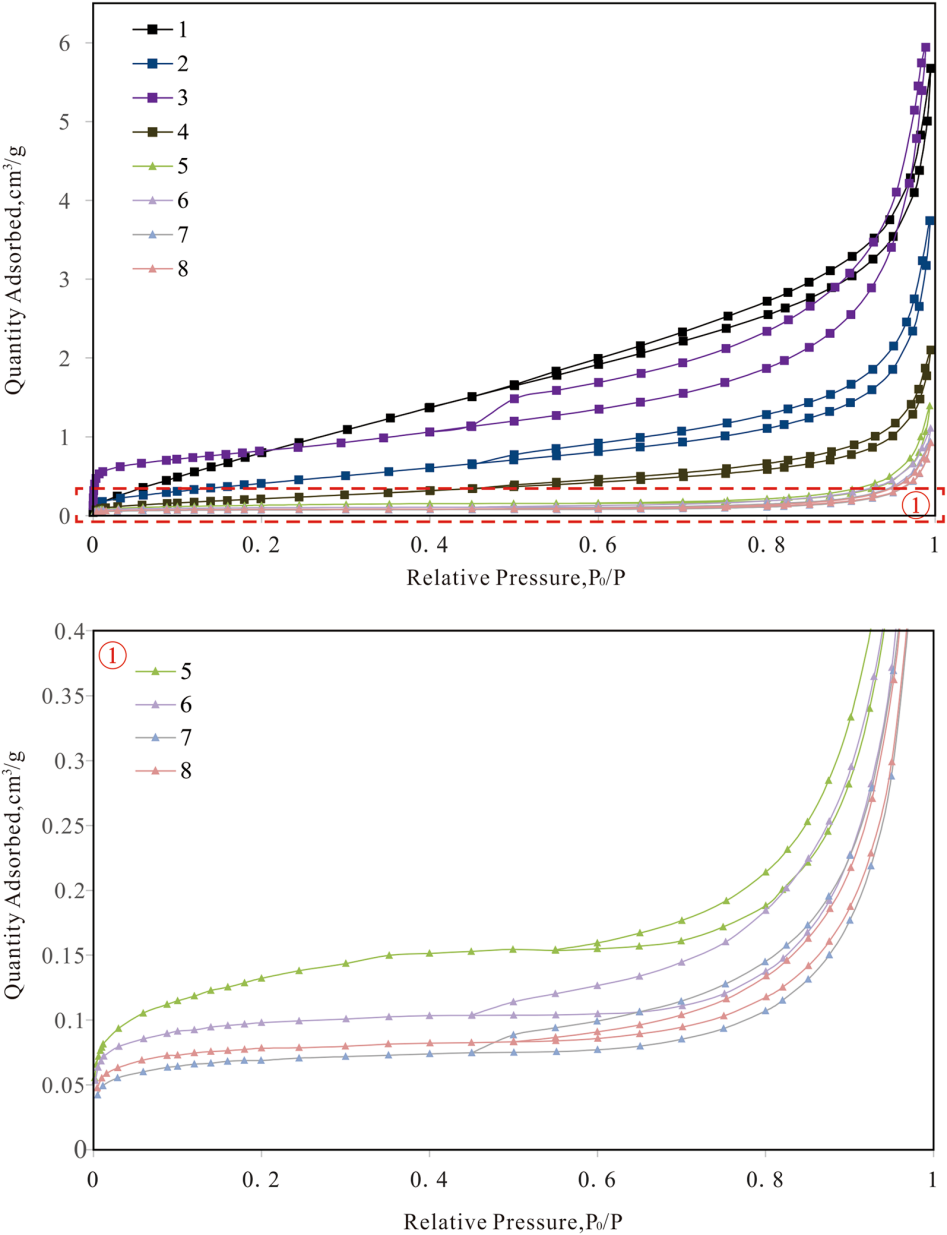
N_2_ gas adsorption/desorption isotherms.

There are many pores less than 100 nm in the sample. For the characterization of pore structures less than 100 nm, nitrogen adsorption–desorption is more accurate than HPMI^23,24^. Therefore, we carried out nitrogen adsorption experiments. Figure 6 presents the adsorption isotherms of the samples. When the relative pressure is very low, the nitrogen exhibits micropore filling and monolayer adsorption. As the relative pressure increases, the nitrogen molecules in the first layer reach saturation, and a relatively obvious inflection point appears in the isotherm adsorption curve (point B in Fig. 6,); then, multilayer adsorption occurs in the metamorphic rocks. As the relative pressure increases, the number of nitrogen molecule adsorption layers gradually increases. When the relative pressure rises to a certain value, the nitrogen gas in the pores starts to condense^23^. None of the samples show a horizontal plateau at a relative pressure close to 1, illustrating that metamorphic samples still contain a range of macropores that cannot be analyzed by nitrogen adsorption experiments^25^. The adsorption quantity in the schist is much higher than that in the mylonite, indicating that the schist has larger pore space than the mylonite.

The type of adsorption isotherm and the shape of the hysteresis loop can indicate the pore type of a porous medium^24^. According to the International Union of Pure and Applied Chemistry (IUPAC) classification, the schist has type II adsorption isotherms, and the mylonite has type III adsorption isotherms. The branch of the adsorption curve and the branch of the desorption curve do not coincide, forming a hysteresis loop that is caused by processes involving adsorption into and desorption from mesopores^21^. According to the IUPAC classification, the schist and mylonite have both H3-type loops and H4-type loops, indicating that the sample pore types are mainly flat, slit-like and ink bottle-shaped.

The results of nitrogen adsorption require the use of different data processing methods, such as the Brunauer–Emmett–Teller (BET)^26,27^, Barrett-Joyner-Hallenda (BJH)^28^ and density functional theory (DFT) models^29^. Nitrogen adsorption can yield three important parameters: specific surface area, average pore diameter and specific pore volume. The BET method is suitable for calculating specific surface area, the BJH method is suitable for calculating average pore diameter, and the DFT method is suitable for calculating specific pore volume^23^. The specific pore volume of the schist is greater than that of the mylonite, which is consistent with the HPMI results. The specific surface area of the schist is also larger than that of the mylonite, while the average pore size is smaller than that of the mylonite (Table 6).

**Table 6:** Nitrogen adsorption parameters of metamorphic rock samples.

Sample ID	Lithology	Specific surface area (m^2^/g)	Average pore diameter (nm)	Specific pore volume (cm^3^/g)
1	Schist	4.3059	5.917	0.0085
2	Schist	1.6785	9.974	0.0049
3	Schist	2.8611	12.683	0.0074
4	Schist	0.8726	10.72	0.00271
Average value of schist		2.4295	9.824	0.00588
5	Mylonite	0.4557	30.635	0.0021
6	Mylonite	0.3062	45.375	0.00168
7	Mylonite	0.218	46.763	0.00024
8	Mylonite	0.781	45.626	0.00028
Average value of mylonite		0.4402	42.100	0.00108

**Figure 7:**
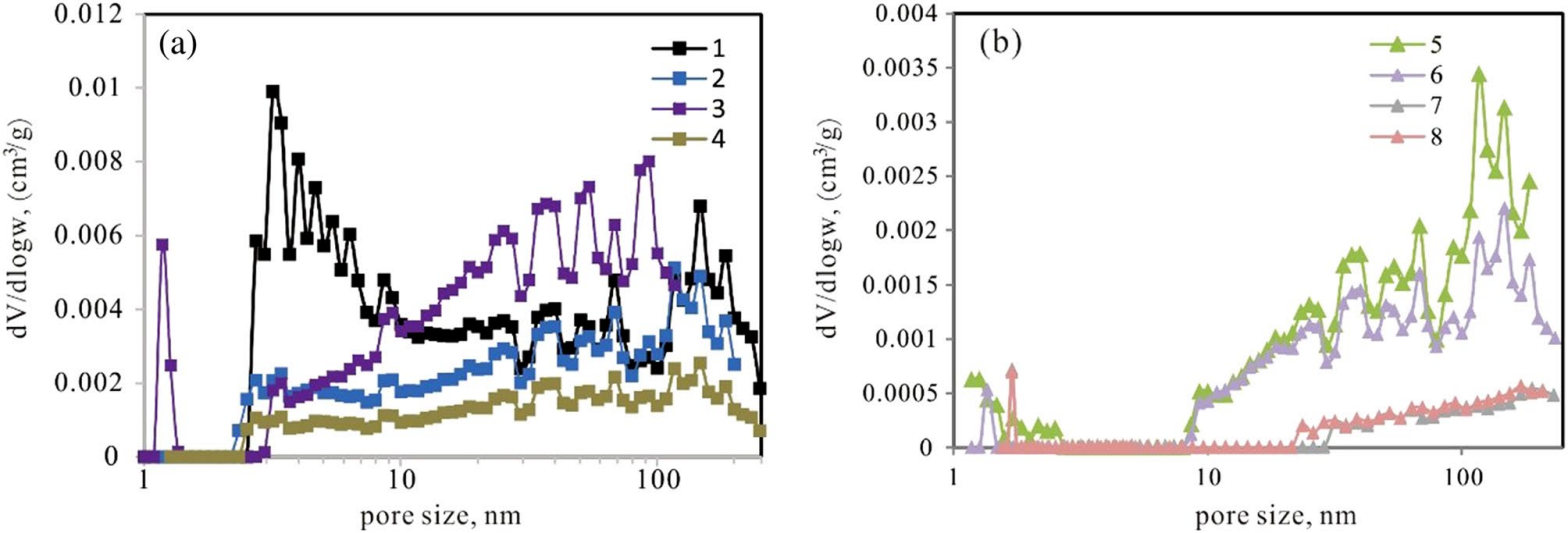
(**a**) PSD analysis of schist samples using DFT methods; (**b**) PSD analysis of mylonite samples using DFT methods.

**Figure 8:**
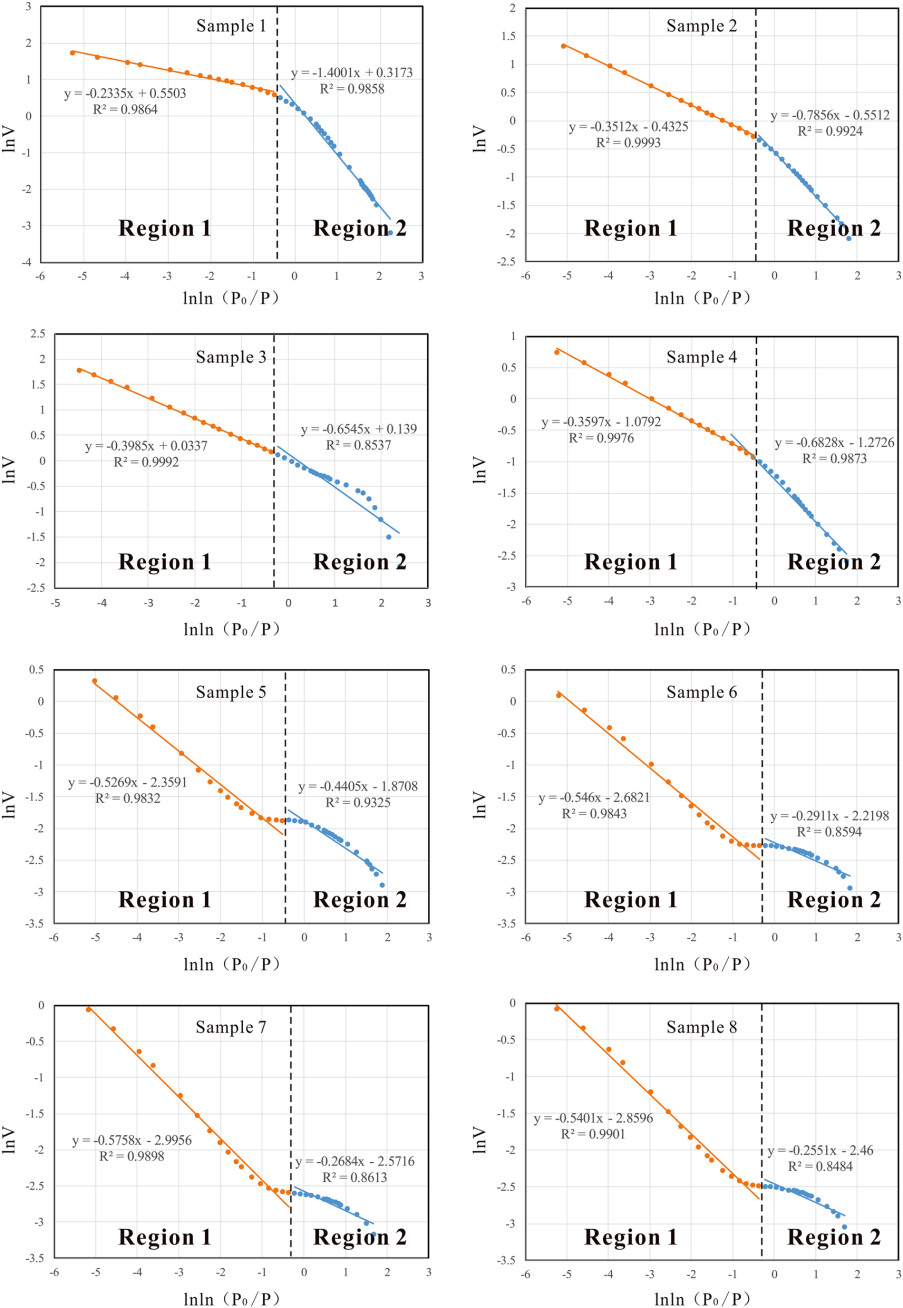
FHH plots for the metamorphic samples.

The BJH model cannot provide a realistic description of pores less than 100 nm ^30^, so we applied the DFT molecular model adsorption branch due to its applicability in determining the PSD of pores less than 100 nm^24,31^. Figures 7 show the PSDs of the samples based on the DFT theory, clearly illustrating the PSD below 100 nm, filling the gap of insufficient detection range and accuracy from HPMI. The PSD curve of the schist exhibits multimodal characteristics, with several maximum pore diameter values larger than 2 nm (Fig. 7). The mylonite has almost no pores smaller than 10 nm, and its pores are mainly distributed between 50 and 100 nm (Fig. 8).

## Discussion

### Pore fractal characteristics

Fractal geometry has a strong ability to describe the irregular or fragmented shapes of natural features as well as other complex objects that traditional Euclidean geometry fails to characterize^32^. The fractal dimension (D) is the key parameter in fractal geometry and provides a systematic approach to quantifying irregular patterns^33,34^. The fractal Frenkel-Halsey-Hill (FHH) model has been proven to be the most effective method for analyzing the fractal behavior of porous media^35^. The FHH model, which has been widely recognized and used^24,35,36^, can be described using the following equation^35^:

$${\text{ln}}\frac{V}{V0} = {\text{constant}} + \left( {D - 3} \right){\text{ ln }}\left( {{\text{ln}}\frac{{{\text{P0}}}}{P}} \right)$$

where V is the quantity of adsorbed nitrogen gas at equilibrium pressure P, V_0_ is the volume of monolayer coverage and P_0_ is the saturation pressure.

According to the equation, on the plot of lnV vs ln (ln (P_0_/P)), the slope of the straight line should be equal to D-3. The FHH plots of metamorphic samples are shown in Fig. 8 and clearly show that the slopes of the FHH graphs of all samples are not uniform, so there is more than one fractal dimension. Based on previous studies^24,35,36^, the nitrogen adsorption isotherm can be divided into two main regions with a relative pressure of 0.5 (P/P_0_ = 0.5), obtaining two fractal dimensions, D1 and D2. Region 1 is the monolayer-multilayer adsorption in which the dominant force is van der Waals, and D1 can be calculated from the slope of the line in region 1. Region 2 is the capillary condensation regime, where the surface tension is the dominant force, and D2 can be calculated from the slope of the line in region 2^37,38^. D1 represents fractals from pore surface areas generated by surface irregularities, while D2 characterizes fractals related to pore structures that are controlled by the composition and pore parameters. Higher fractal dimension D1 correlates to more irregular surfaces that provide more space for CH_4_ adsorption. Higher fractal dimension D2 represents more significant heterogeneity of pore structure and higher liquid/gas surface tension that reduce CH_4_ adsorption capacity^35^. The calculation results (Table 7, detailed data and calculation process can be found in the Supplementary Information) show obvious regularity. The D1 of the schist is lower than that of the mylonite; the average D1 of the schist is 2.1193, while the average D1 of the mylonite is 2.6782, indicating that the mylonite has more irregular surfaces. The D2 of the schist is higher than that of the mylonite; the average D2 of the schist is 2.6643, while the average D2 of the mylonite is 2.4543, indicating that schist has a more significant pore heterogeneity of pore structure.

**Table 7:** Calculation results for fractal dimensions.

Sample ID	Lithology	D1	D2
1	Schist	1.5999	2.7665
2	Schist	2.2144	2.6488
3	Schist	2.3455	2.6015
4	Schist	2.3172	2.6403
Average value of schist		2.1193	2.6643
5	Mylonite	2.5595	2.4791
6	Mylonite	2.7089	2.4540
7	Mylonite	2.7316	2.4242
8	Mylonite	2.7489	2.4599
Average value of mylonite		2.6872	2.4543

### Storage capability

The natural gas stored in the reservoir has two phases: free gas and adsorbed gas^39^.

Free gas is mainly stored in pores and fractures of rocks, and its storage capacity is mainly determined by pore volume^39^. Except for sample 4, the pores of all samples less than 1 micron (nano scale) account for about 90% of the total pore volume (Table 5). Therefore, pores less than 1 micron are the primary locations for free gas storage in the metamorphic rock samples, controlling the storage capability for free gas. Adsorbed gas storage potential is primarily decided by pore surface area of and clay minerals^40^. The clay mineral content and specific surface area of schist are higher than mylonite, so schist has larger adsorption gas storage capacity.

### Formation process of the metamorphic rock reservoir

The U–Pb zircon dating results from our research team (Table 8) reveal that the concordant age of the chlorite schist is 131.3 ± 8.5 Ma, indicating that the andesitic protolith formed during the deposition of the upper Shahezi Formation and lower Yingcheng Formation in the Cretaceous. The concordant age of unmylonitized granite in the upper part of the LT2 well is 160.7 ± 1.9 Ma, which is Late Jurassic; the concordant age of granitic mylonite in the LT1 and LT2 wells is 264.2 ± 2.6 Ma, indicating that the formation of the granitic protolith occurred in the middle-late Permian.

**Table 8:** Reservoir chronological results for the central paleo-uplift belt.

Lithology	Protolith	Chronology	Formation time of the protolith
Chlorite schist	Andesite	131.3 ± 8.5 Ma	Late Shahezi period-early Yingcheng period in Cretaceous
Granite		160.7 ± 1.9 Ma	Late Jurassic
Mylonite	Granite	264.2 ± 2.6 Ma	middle-late Permian
Mica schist	Clastic rock	275.2 ± 2.6 Ma	early-middle Permian

The concordant age of the the mica schist in Well LT1 is 275.2 ± 2.6 Ma, indicating that the diagenesis of its clastic protolith occurred no earlier than 275.2 Ma (early Permian). Because the upper granite formed in the middle-late Permian, the formation of the clastic rock should have occurred earlier than the middle-late Permian, so the formation time of the clastic rock can be constrained to the early-middle Permian.

**Figure 9:**
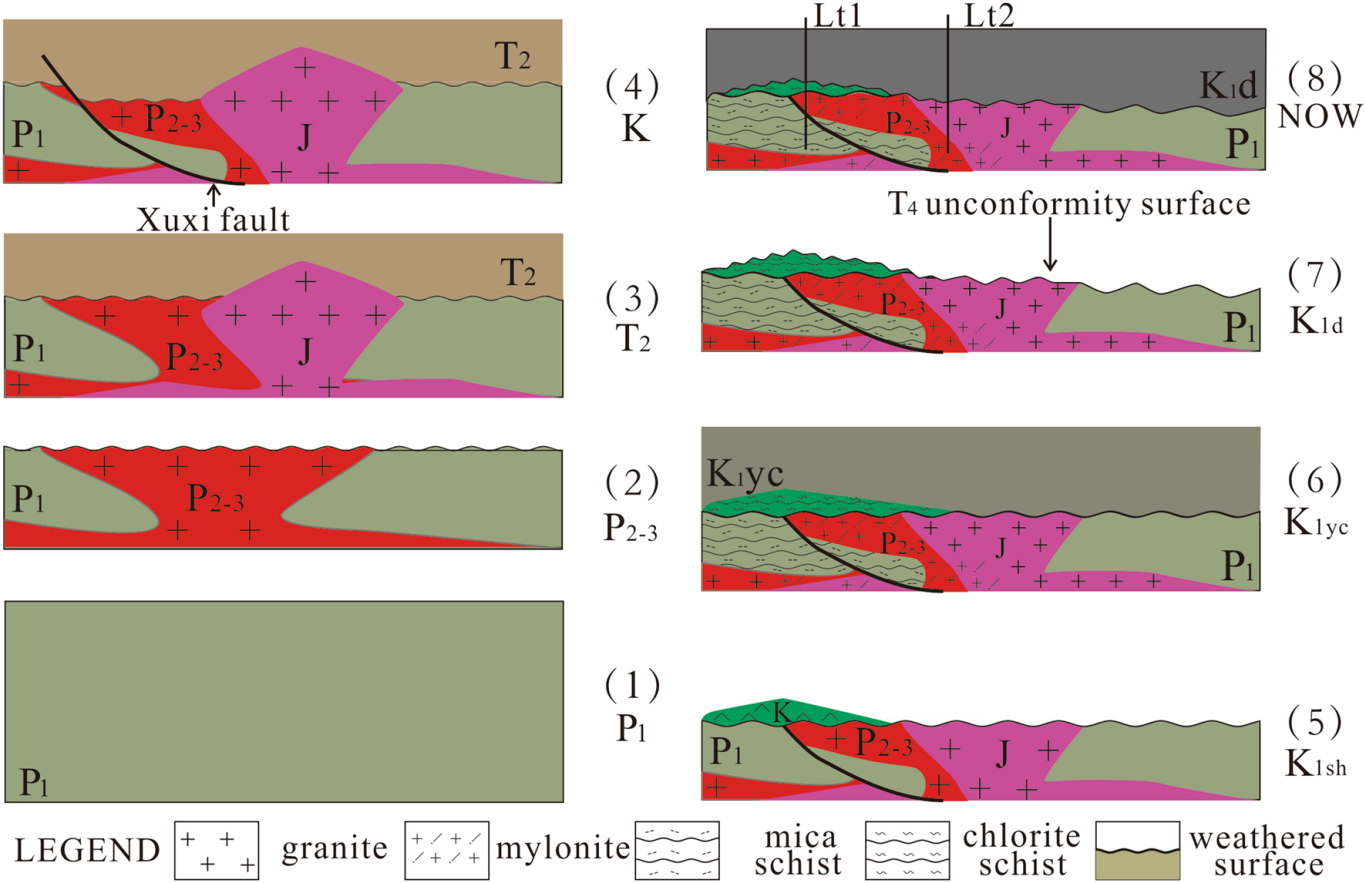
Inferred formation process of the metamorphic rock reservoir (This figure was drawn by CorelDRAW Graphics Suite 2019, vision number: 21.3.0.755, url:https://www.corel.com/cn).

According to the structural background of the Songliao Basin and the four chronological test results, we hypothesize that the formation process of the metamorphic rock reservoir in the central paleo-uplift belt can be divided into the following four stages (Fig. 9):

1. Formation and weathering of Permian protoliths

In the early-middle Permian, thick marine strata were deposited (Fig. 9-1), and granitic magma then intruded in the middle-late Permian to form granite, forming the protolith of the mylonite. From the end of the Permian to the Early Triassic, the collision of the northeastern block and the North China Craton occurred along the Sauron-Siramulun-Changchun suture zone; this process uplifted the crust, and the strata underwent erosion (Fig. 9-2)^41^.

(2) Formation and weathering of Mesozoic protoliths

Previous studies have found Triassic strata in the Songliao Basin^42^. The Middle Triassic strata began to be deposited, and the Late Jurassic granitic magma later intruded (Fig. 9-3). In the Cretaceous period, when the Huoshiling Formation was deposited, during the low-angle subduction of the Pacific Plate towards the Asia-Europe Plate, basement faults became active^43^, and the Xuxi fault began to form (Fig. 9-4)^44^. During the Cretaceous Huoshiling-Shahezi depositional period, weathering and erosion occurred. During the late Shahezi-early Yingcheng depositional period in the Cretaceous, lava erupted to form andesite, representing the protolith of the chlorite schist (Fig. 9-5).

(3) Metamorphic stage

The development of the basement reservoir in the central paleo-uplift zone of the Songliao Basin was related to the activity of the Xuxi fault^17^. Large-scale activity of the Xuxi fault occurred during the Shahezi-Yingcheng depositional period^44^, and the surrounding rocks were metamorphosed. Andesite in the upper part underwent both brittle deformation and ductile deformation under strong stress. At the same time, chlorite was produced by alteration of hornblende and formed the chlorite schist. The granite in the middle part also experienced brittle and ductile deformation but more ductile deformation. The clastic rocks in the lower part were buried deeper, and the increase in temperature triggered regional metamorphism. The mud was transformed into biotite, muscovite, garnet and chlorite, and the feldspar and quartz particles became directionally aligned, forming the mica schist. From shallow to deep, the degree of ductile deformation gradually increased, and the deep rock layer experienced regional metamorphism, which conforms to the behavior of dynamic metamorphic rock (fault rock)^45^. The Late Jurassic granite was not metamorphosed because it was far from the fault (Fig. 9-6).

(4) Weathering and deep burial

There is an unconformity between the Yingcheng Formation and Denglouku Formation in the Cretaceous, which is called the T4 unconformity surface. In the Yingcheng-Denglouku period, the strata underwent weathering and erosion. Because the Yingcheng Formation had a weak resistance to weathering and erosion, all of these rocks were removed. However, the chlorite schist is relatively resistant to weathering and erosion and was only partially removed, forming a weathering crust (Fig. 9-7). Subsequently, the overlying Cretaceous Denglouku Formation was deposited (Fig. 9-8).

### Factors controlling microscopic pore characteristics

1. Effect of metamorphism on microscopic pore characteristics

For andesite (the protolith of the chlorite schist), dynamic metamorphism can cause deformation and fracturing, increasing the reservoir space, while fine-grained chlorite is produced by alteration of hornblende and fills previously formed pores and fracture pores; thus, dynamic metamorphism has both constructive and destructive effects on the reservoir.

For granite (the protolith of the mylonite), dynamic metamorphism causes the originally dense granite to break and crack, having a constructive effect on the reservoir.

For clastic rocks (protoliths of the mica schist), regional metamorphism causes the minerals to recrystallize and directionally align, reducing the original pores and having destructive effects on the reservoir.

(2) Effect of structural location on microscopic pore characteristics

The influence of the structural position on microscopic pore characteristics is mainly reflected in the reservoir space derived from physical weathering. The chlorite schist and mylonite in the LT1 well and Late Jurassic granite in the LT2 well are weathered and eroded, so they all have weathering fractures, while the mica schist was below the surface, so no weathering fractures are observed.

(3) Effect of the protolith on microscopic pore characteristics

Many microscopic pore characteristics are related to the protolith characteristics.

Andesite is formed by the eruption and rapid cooling of magma on the surface. Due to different volatiles, the vesicles in andesite are unevenly distributed, and phenocrysts are unevenly distributed in the matrix. The rock can also undergo varying degrees of weathering and leaching at the surface. Therefore, andesite is not homogeneous. Clastic rocks are formed by cementing various types of debris with different grain sizes; thus, they are also not homogeneous. In contrast, granite forms underground where the temperature is higher. The temperature decreases slowly, and it takes a long time for granite to crystalize, so it is relatively homogeneous. Judging from the results, both HPMI and fractal algorithms reveal that the schist is more significant heterogeneous than the mylonite.

The protoliths of the schist, whether andesite or clastic rock, were loose and porous, while the protolith of the mylonite was granite, which is very dense and basically nonporous. The HPMI experiments show that the schist has a higher porosity than the mylonite, and the nitrogen adsorption tests show that the adsorption quantity and specific pore volume of the schist are much largerthan those of the mylonite. Although metamorphism reduced the pore space in the schist and increased the pore space in the granite, the original pore space of the protoliths largely controls the final pore space.

Generally, among the three kinds of protoliths, argillaceous siltstone has the most clay minerals, andesite has the second highest amount, and granite has the lowest amount of clay minerals. According to the SEM observations, the mica schist has the most clay minerals, including biotite, montmorillonite and chlorite. The chlorite schist contains a large amount of chlorite, and the mylonite contains only a very small amount of chlorite.

Quartz and feldspar are both brittle granular minerals that are prone to cracking under tectonic stress^12^, while dark-colored minerals are more elastic, have high ductility and are not prone to cracking^13^. Therefore, microfractures have developed in the mylonite, but microfractures cannot be found in the schist.

In addition, the protoliths controlled the metamorphic histories of the rocks to some extent. The granite in the middle part and the clastic rocks in the lower part are continuous vertically, and both formed in the Permian, but their metamorphic styles are different. The clastic rocks contain many impurities, such as matrix material, mica and chlorite debris, which are prone to alteration. In contrast, the granite mineral composition is basically quartz and feldspar, which are simple, relatively stable and not prone to alteration. Temperature was the dominant factor in the metamorphism of the schist, which overall experienced regional metamorphism, while stress was the dominant factor in the metamorphism of the mylonite and caused partial dynamic metamorphism.

In summary, the microscopic pore characteristics of the metamorphic rock reservoir are controlled by the type of metamorphism, structural location and protolith, among which the protolith was the most important controlling factor.

## Conclusions

The combination of qualitative descriptions and quantitative analysis was used to comprehensively analyze the pore characteristics of metamorphic rock reservoirs in the central paleo-uplift belt of Songliao basin. The main rock types are chlorite schist, mica schist and mylonite, and the reservoir space can be divided into intergranular pores, dissolved pores, structural microfractures and weathering microfractures. The PSDs of the schist and mylonite are different. Compared with the mylonite, the schist has larger reservoir space, more significant heterogeneity, smaller pore size, largerspecific surface area and larger adsorbed gas storage capacity, which has certain guiding significance for the exploration and development of metamorphic rock reservoirs. The microscopic pore characteristics of the metamorphic rock reservoir are controlled by the metamorphism, structural location and protolith, among which the protolith is the most important controlling factor.

## Supplementary Information

Supplementary Information.
